# Multimodal MR Images-Based Diagnosis of Early Adolescent Attention-Deficit/Hyperactivity Disorder Using Multiple Kernel Learning

**DOI:** 10.3389/fnins.2021.710133

**Published:** 2021-09-14

**Authors:** Xiaocheng Zhou, Qingmin Lin, Yuanyuan Gui, Zixin Wang, Manhua Liu, Hui Lu

**Affiliations:** ^1^Shanghai Jiao Tong University-Yale Joint Center for Biostatistics and Data Science, Shanghai Jiao Tong University, Shanghai, China; ^2^Department of Bioinformatics and Biostatistics, School of Life Science and Biotechnology, Shanghai Jiao Tong University, Shanghai, China; ^3^Department of Developmental and Behavioral Pediatrics, Shanghai Children's Medical Center, School of Medicine, Shanghai Jiao Tong University, Shanghai, China; ^4^MoE Key Lab of Artificial Intelligence, AI Institute, Shanghai Jiao Tong University, Shanghai, China; ^5^Department of Instrument Science and Engineering, School of EIEE, Shanghai Jiao Tong University, Shanghai, China; ^6^Center for Biomedical Informatics, Shanghai Engineering Research Center for Big Data in Pediatric Precision Medicine, Shanghai Children's Hospital, Shanghai, China

**Keywords:** early adolescent, attention-deficit/hyperactivity disorder, multimodal MR images, disorder diagnosis, multiple kernel learning, structural MRI, DTI, resting-state functional MRI

## Abstract

Attention-deficit/hyperactivity disorder (ADHD) is one of the most common brain diseases among children. The current criteria of ADHD diagnosis mainly depend on behavior analysis, which is subjective and inconsistent, especially for children. The development of neuroimaging technologies, such as magnetic resonance imaging (MRI), drives the discovery of brain abnormalities in structure and function by analyzing multimodal neuroimages for computer-aided diagnosis of brain diseases. This paper proposes a multimodal machine learning framework that combines the Boruta based feature selection and Multiple Kernel Learning (MKL) to integrate the multimodal features of structural and functional MRIs and Diffusion Tensor Images (DTI) for the diagnosis of early adolescent ADHD. The rich and complementary information of the macrostructural features, microstructural properties, and functional connectivities are integrated at the kernel level, followed by a support vector machine classifier for discriminating ADHD from healthy children. Our experiments were conducted on the comorbidity-free ADHD subjects and covariable-matched healthy children aged 9–10 chosen from the Adolescent Brain and Cognitive Development (ABCD) study. This paper is the first work to combine structural and functional MRIs with DTI for early adolescents of the ABCD study. The results indicate that the kernel-level fusion of multimodal features achieves 0.698 of AUC (area under the receiver operating characteristic curves) and 64.3% of classification accuracy for ADHD diagnosis, showing a significant improvement over the early feature fusion and unimodal features. The abnormal functional connectivity predictors, involving default mode network, attention network, auditory network, and sensorimotor mouth network, thalamus, and cerebellum, as well as the anatomical regions in basal ganglia, are found to encode the most discriminative information, which collaborates with macrostructure and diffusion alterations to boost the performances of disorder diagnosis.

## 1. Introduction

Attention-deficit/hyperactivity disorder (ADHD) has become one of the most common neurobehavioral disorders among children (Polanczyk et al., 2015). In 2016, 9.4% of children and adolescents aging 2–17 in the United States had ever been diagnosed with ADHD, 89.4% of which still kept the diagnosis at present (Danielson et al., 2018). Untreated ADHD can cause substance abuse and tremendous academic, social, and financial/employment burdens on the individual and family (Hamed et al., 2015), reflecting the importance of diagnosing and treating the disorder. Medication and behavioral intervention have been demonstrated to ameliorate the conditions of ADHD patients (Hoogman et al., 2019). To afford the affected ability to achieve their full potential in school or at home, ADHD is screened for and diagnosed as early as possible (Hamed et al., 2015). However, the most advanced standard of ADHD diagnosis is symptom-based, according to the Diagnostic and Statistical Manual of Mental Disorders, the 5th edition (Wolraich et al., 2019) (DSM-5), relying on the questionnaires collected from the parents or caregivers for young children. The multi-source reports, however, are subjective and usually cause inconsistency and bias. Therefore, the diagnosis of ADHD requires objective and quantizable evidence.

The advancement of high-resolution brain imaging technologies and high-throughput computing makes it possible to build a computer-aided diagnostic system for mental health disorders based on the quantitative features extracted from the images. The brain imaging technologies, such as magnetic resonance and computed tomography, have shown brightening perspectives to reveal the underlying pathophysiology of ADHD. MRI becomes the ideal technology to study brain diseases for its high-resolution inner tissue imaging capability. Structural MRI (sMRI), diffusion MRI (dMRI), and functional MRI (fMRI) have been widely applied in ADHD studies in recent years to explore the quantizable features indicating various-level brain alterations in cortical and subcortical measures, such as morphometric traits, diffusion properties, and functional connectivity (FC). However, these findings depend on the hypothesis tests between the experiment and control groups with small sample sizes, limiting the power for unveiling the relationship between features and building usable automatic diagnosis (Arbabshirani et al., 2017).

The growing minable image-based features motivated radiomics (Gillies et al., 2016), an emerging efficient paradigm aiming at quantitative image analytics and automatic diagnosis through recognizing intricate patterns among the high-dimensional traits from images (Hosny et al., 2018; Ibrahim et al., 2020). In this medical image analysis framework, the collected image data are segmented into regions of interest (ROIs). The features of multiple levels, including the intensity distribution, shape, and texture, are extracted from these ROIs and qualified. Subsequently, the predictive models are built on the features to support the decision-making for diagnostic or prognostic. The state-of-art machine learning and deep learning approaches have triggered vitality in the medical image recognition community (Hosny et al., 2018). The radiomics practices in automatic ADHD diagnosis have sprouted in the past 10 years along with the release of the ADHD-200 consortium (Milham et al., 2012; Bellec et al., 2017), the hitherto largest multimodal dataset concentrating on ADHD. ADHD-200 provides the sMRI and resting-state fMRI (rsfMRI) images and the personal characteristic features of 362 patients and 585 healthy people aging 7–27 aggregated from 17 different studies conducted across eight various sites. Based on the dataset, researchers extracted features from MRI data within clustered voxels or predefined ROIs and built machine learning algorithms for classification task. Support vector machine (SVM) has become the most popular classification model (Arbabshirani et al., 2017; Sakai and Yamada, 2019; Biswas et al., 2020; Lohmann et al., 2020) for its outperformance in multivariable data using appropriate kernel functions. Ghiassian et al. combined the sMRI or fMRI features with the characteristic features and applied RBF-SVM as classifiers for predicting ADHD (Ghiassian et al., 2016). However, most studies only use the sMRI or/and rsfMRI to diagnose ADHD; rare practice considered dMRI features, which is believed to reveal the critical microstructure abnormality in ADHD's brain (van Ewijk et al., 2012; Lei et al., 2014; Gehricke et al., 2017), into the predictive model. Moreover, the current multimodal studies directly concatenated all of the features to a large vector and fed it to the classifiers (Sun et al., 2018; Luo et al., 2020). Though the improved performance suggests that multiple modalities have complementary information for classification, the features encoding minor information and the curse of high dimension might impair such a strategy.

The current study applies multiple kernel learning (MKL) framework to fuse the sMRI, rsfMRI, and DTI features collected from the Adolescent Brain and Cognitive Development (ABCD) study and predict ADHD diagnosis. MKL, a commonly used model-based fusion strategy in multimodal learning (Baltrusaitis et al., 2019), has been verified to effectively integrate the heterogenetic source information by assigning specified kernels and weight to distinct modalities (Gönen and Alpaydın, 2011). It has been widely implemented in visual object recognition (Bucak et al., 2014), remote sensing (Niazmardi et al., 2018), hyperspectral image classification (Gu et al., 2017), and medical image fusion and classification (Wen et al., 2017; Schrouff et al., 2018; Wani and Raza, 2018). Our study focuses on comorbidity-free early adolescent patients aging 9–10. To solve the high-dimension problem, we apply Boruta, a random-forest-based feature selection method, to choose all the relevant features of ADHD. This technique is appropriate for the highly correlated network of human brain structures. We hypothesize that the MKL framework, the kernel-level multimodal fusion strategy, would achieve higher performance in the task of ADHD diagnosis based on multimodal imaging data, and the discovered all relevant predictors from multimodal MRI would help unveil the underlying mechanism of ADHD.

## 2. Materials and Methods

### 2.1. Dataset Description and Participants

ABCD study is the most extensive long-term study of brain development and child health in the United States, which recruited 11,878 children ages 9–10 in 21 research sites across the United States. This project sampled children through schools based on US society's demographic profile (Garavan et al., 2018) and keeps following them up through their adolescence to early adulthood for tracking their biological and behavioral development. Various MR imaging data, genomics data, and the scales and questionnaires of mental health, physical health, demographics, and neurocognition are deposited (Barch et al., 2018) and released yearly. The image data that children underwent include T1- and T2-weighted MRI, DTI, rsfMRI, and three task-based fMRI scans. After the image acquisition, these multimodal MR images were uploaded to the Data Analysis, Informatics, and Resource Center (DAIRC) of the ABCD Study. Then there, the quality control, image preprocessing, measuring based on multi-atlases, and tabulating were completed in a standard pipeline. The published paper (Hagler et al., 2019) from the ABCD team has stated the acquisition, scanning parameters, and processing pipelines in detail. For the brevity of the main text, we describe the images processing steps and acquisition parameters concisely in Supplementary Material. The relatively narrow and early age span, consistent diagnostic criteria across all the sites, and multimodal data sources characterize the ABCD study with the potential of studying the mental disorders' development trajectory. This work concentrates on the tabulated multiple-type image-based features, including the quantitative brain properties extracted from sMRI (T1/2 weighted parts), rsfMRI, and DTI, of the baseline year in release 2.0.1 (Jernigan et al., 2019) for further analysis of ADHD.

To label the ADHD patients, we reviewed the ABCD Parent Diagnostic Interview scale for DSM-5 Full of K-SADS of the baseline year for ADHD diagnosis. The subjects under the following conditions were excluded: with missing values in MRI scanning and the covariables, the left-handed, ever-experienced traumatic brain injury with loss of consciousness, the main comorbidities of ADHD (Homer et al., 2000; Wolraich et al., 2019), covering tic disorders, emotional disorders (phobia, anxiety disorders, disruptive mood dysregulation disorders, depression disorders, and bipolar disorders), autism spectrum, psychotic disorders, post-traumatic stress disorder, oppositional defiant disorders, and conduct disorders for reducing the influence of covariables as much as possible. According to the suggestion in the Fix Note of the ABCD study, we censored the subjects that detected clinical referrals or did not pass the quality control procedure of T1w, T2w, dMRI, and rsfMRI. The subjects scanned on Phillips machines were excluded due to incorrect post-processing of fMRI data noticed in the officially released issue. Without any ADHD-related diagnosis and symptoms or any other mental health diagnosis, the children were chosen to match the race and sex with ADHD as the typical controls (TC). The workflow in Supplementary Figure 2A shows the number of subjects that are considered after applying various exclusion criteria, and Supplementary Figures 2B,C with Supplementary Tables 1, 2 list the percentage and count of subjects filtered by each item within the groups. The demographic information of the matched groups of ADHD and TC is summarized in Table 1. There are no statistically significant differences in the main covariates of age, gender, and race.

**Table 1:** Demographic description.

		**Control**	**Case**	***P*-value**
Total		116	116	–
Sex	Female	45	45	–
	Male	71	71	
Race	White	80	80	–
	Hispanic	13	13	
	Black	10	10	
	Asian	12	12	
	Other	1	1	
Age (month)		118.9 (7.7)	118.4 (7.7)	0.59

*The values are denoted as mean (standard deviation)*.

### 2.2. Imaging Measures

In our study, the cortical ROIs were labeled with structural-based atlas (Desikan-Killiany atlas, Desikan et al., 2006 for sMRI and DTI, major white matter tracts' AtlasTrack Hagler et al., 2009 for DTI), genetic-based atlas [fuzzy-cluster parcels (Chen et al., 2012) for sMRI], tract fiber atlas, and functional connectome atlas [Gordon parcellations (Gordon et al., 2016) for fMRI], and the subcortical regions were labeled with atlas-based segmentation (Fischl et al., 2002) (for sMRI, DTI, and fMRI). These brain atlases were frequently used in the corresponding modalities so that the accumulated publications would offer direct evidence supporting our findings.

In this work, the macrostructural property of the brain refers to the morphometry and image intensity measures extracted from sMRI. Morphometric measures consist of cortical volume, thickness, area, and sulcal depth, and subcortical volume. Image intensity measures include intensity properties of T1w, T2w, and T1w and T2w cortical contrast. The alterations in morphometry and intensity indicate the abnormal development and changed composition of brain tissue (Kotov, 2017; Bloem et al., 2018), which are common features of many neurological diseases. Cortical contrast has also been confirmed to serve as a sensitive cortical marker of brain development and psychopathology (Lewis et al., 2018; Norbom et al., 2019).

The major white fibers' volume and four measures accessing water diffusion in cortical and subcortical tissues, including fractional anisotropy (FA) and mean, longitudinal, and transverse diffusivity (MD, LD, and TD), were extracted from DTI to indicate microstructural tissue properties. The FA reflects directionality estimation in tissue characteristics like myelination and fiber density, and MD, LD, and TD characterize the diffusion magnitude in distinct directions (Alexander et al., 2007). These measures have been applied in image-based brain diseases analysis (van Ewijk et al., 2012; Lei et al., 2014; Gehricke et al., 2017).

For rsfMRI, the candidate features are functional connectivity of cortical function network and subcortical regions. The average correlation values were calculated between paired cortical function network ROIs and then transformed to z-score, representing the strength of FC (Van Dijk et al., 2010). Similarly, the FC between each network and each subcortical region was collected as well. FC reflects a straightforward, observational measure of functional relationships between the target networks, which has been a universal tool to analyze ADHD (Lin and Roth, 2017; Samea et al., 2019; Sörös et al., 2019).

The study considered 2,704 candidate predictors collected from the tree modalities (1,184 from sMRI, 1,182 from DTI, and 338 from rsfMRI) and modeled them to remove the scanners' fixed bias of batch effect with Combat (Johnson et al., 2007), the effectiveness of which has been confirmed in MRI-derived features in the recent years (Fortin et al., 2017, 2018; Yu et al., 2018). Details of the candidate predictors were listed in Supplementary Table 3, and the model and formulas of Combat were described in the Supplementary Materials.

### 2.3. Multimodal Feature Selection and Assessment

Boruta (Kursa and Rudnicki, 2010) is an outperformed all-relevant feature selection method based on the random forest (RF). Boruta concatenates shuffled features, named shadow features, with the original data and builds an RF classifier. The Gini impurity's decrease in each base decision tree indicates the contribution of features to classification. RF summarizes and normalizes the score for each feature. The original features that achieve higher scores than the highest in the shadow features are marked. The shuffling-scoring-marking procedures are iteratively executed, and a series of features set is selected. A binomial distribution is established to test if a given feature significantly scores higher than any random one until all the original features are confirmed relevant or rejected. *P*-value correction for multiple testing is considered. Comparing with the minimal optimal feature selection methods that try to find a compact feature subset to minimize the error of a classifier, all-relevant methods manage to pick up all features coding information usable for classification, the property of which is suitable for highly intercorrelated biomedical data (Kursa, 2014). It has been applied in various contexts, including ADHD image-based predictors finding (Sun et al., 2018), and achieved outstanding performance in robustness, efficiency, and effectiveness (Kursa, 2014; Speiser et al., 2019).

### 2.4. Multimodal Fusion and Disease Classification

SVM is a general framework of classification or regression based on the kernel method. Linear or non-linear kernel applied, SVM has rendered itself outperformance with other models in various domains. According to the dual problem of SVM, we supposed the optimized objective of the multiple kernel SVM as the following equation:


(1) $$\begin{array}{l} \underset{\alpha ,w}{\text{min}}\frac{1}{2}\overset{N}{\underset{i=1}{\sum }}\overset{N}{\underset{j=1}{\sum }}\alpha_{i}\alpha_{j}y_{i}y_{j}\underset{m\in M}{\sum }w_{m}k_{m}\left(x_{i}^{m},x_{j}^{m}\right)-\overset{N}{\underset{i=1}{\sum }}\alpha_{i} \end{array}$$

$$\begin{array}{l} s.t.\overset{N}{\underset{i=1}{\sum }}\alpha_{i}y_{i}=0,0\leq \alpha_{i}\leq C,i=1,2,\ldots ,N \end{array}$$

where $\{{\left({\left\{x_{i}^{m}\right\}}_{m\in M},y_{i}\right\}}_{i=1}^{N}$ are the samples with multiple feature sets ${\left\{x_{i}^{m}\right\}}_{m\in M}$ and label *y*_*i*_ of the training set with sample size *N*, *k*_*m*_(·, ·) represents a positive definite Gram matrix or kernel, *w*_*m*_ is the weight assigned to the corresponding kernel with the constraint $\underset{m\in M}{\sum }w_{m}=1$, the ${\left\{\alpha_{i}\right\}}_{i=1}^{N}$ are the Lagrange multipliers introduced by dual algorithm, and *C* is the penalty assigned to misclassified samples. We solved the problem by iteratively searching the *w*_*m*_ and optimizing the ${\left\{\alpha_{i}\right\}}_{i=1}^{N}$. The decision function is given as Equation (2):


(2) $$\begin{array}{l} f\left(x\right)=sign\left(\overset{N}{\underset{i=1}{\sum }}\alpha_{i}^{*}y_{i}\underset{m\in M}{\sum }w_{m}^{*}k_{m}\left(x_{i}^{m},x^{m}\right)+b^{*}\right) \end{array}$$

where ${\left\{x^{m}\right\}}_{m\in M}$ is the sample with *M* feature sets in the testing set, and ${\left\{\alpha_{i}^{*}\right\}}_{i=1}^{N}$, ${\left\{w_{m}^{*}\right\}}_{m\in M}$ and b^*^ are the optimized parameters, $b^{*}=y_{j}-\overset{N}{\underset{i}{\sum }}\alpha_{i}^{*}y_{i}\underset{m\in M}{\sum }w_{m}^{*}k_{m}\left(x_{i}^{m},x_{j}^{m}\right)$.

Our study set the searching stride of *w*_*m*_ to 0.1 for the feature sets *M*, including DTI, rsfMRI, and sMRI. The kernel functions include linear kernel and radial basis function (RBF) kernel, the most popular kernel function for continuous features for its infinite kernel feature space to exploit the non-linear relationship. The kernel function implicitly projects the original data to a higher-dimensional space and measure the distance (or similarity) between two samples in that space. Classification performance is promoted via choosing a kernel suitable for specific data. The data from different modalities, however, are probably ideal for distinct kernel functions or different parameters. By linearly combining the kernels from different feature spaces, the integration of heterogenetic sources can be achieved at the kernel level. In the MKL framework, additional restrictions, such as setting any *w*_*m*_ = 0, can be given to tackle with modality missing.

Single-kernel SVMs were applied to each modality as the unimodal baseline and the directly concatenated multimodal features as the early fusion baseline. As a commonly used classifier in mental disorder studies, RF was implemented in both settings as a baseline.

### 2.5. Classifier Training and Cross-Validation

**Figure 1:**
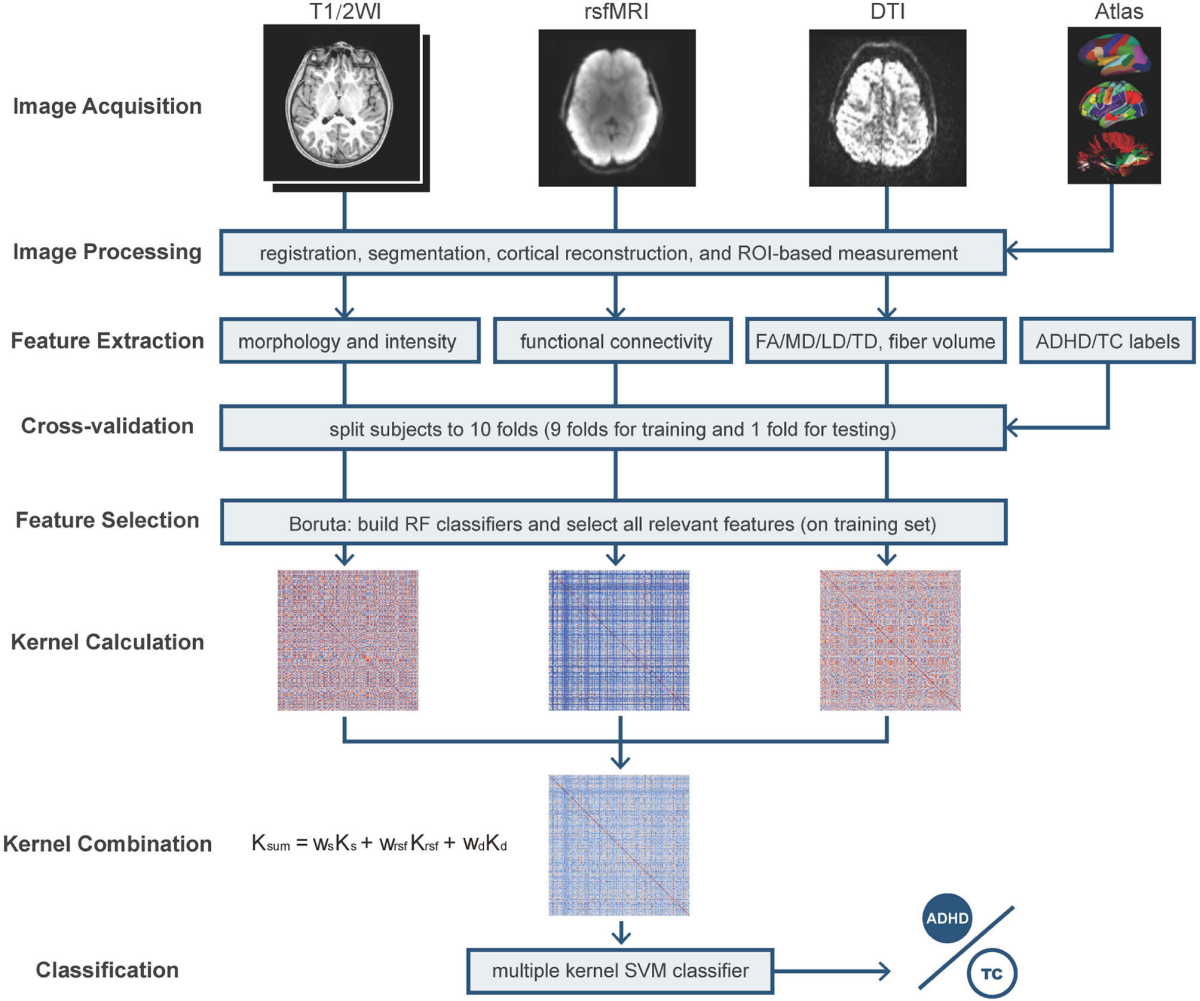
The pipeline of feature extraction and cross-validation Multiple Kernel Learning (MKL) classification. T1/2WI, T1 weighted imaging and T2 weighted imaging; rsfMRI, resting-state fMRI; DTI, diffusion tensor imaging; FA, fractional anisotropy; MD, mean diffusivity; LD, longitudinal diffusivity; TD, transverse diffusivity; RF, random forest; SVM, support vector machine. The used atlases include Desikan–Killiany atlas, fuzzy-cluster parcels, subcortical regions, AtlasTrack, and Gordon parcellations; the morphological features include subcortical region volume, cortical volume, thickness, area, and sulcal depth.

**Figure 2:**
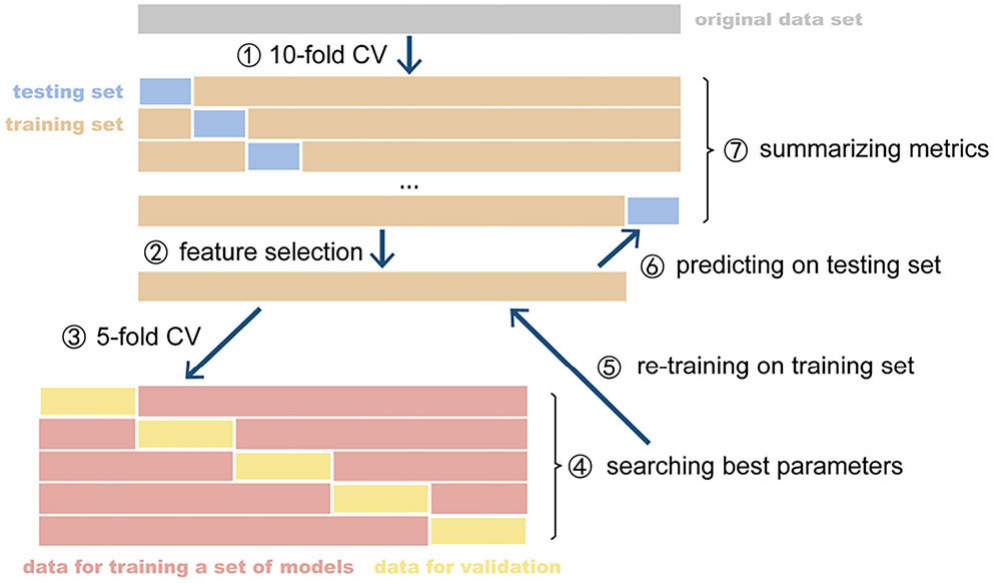
The CV and nested CV processes. CV, cross-validation. The parameter optimization of model was implemented on the inner CV and retrained optimized model was evaluated on the outer CV. The metrics of all fold were summarized as the expected performance of the model.

As shown in Figure 1, the 10-fold cross-validation was manipulated on the three modalities using the same division of subjects every fold, respectively. In an individual iteration, we performed Boruta selection on the training set to pick up all relevant features. The dataset of each modality shrunk to a smaller dimension. We constructed the kernel matrix for every modality and applied optimistic weight to fuse them by grid-searching. In each iteration, as shown in Figure 2, five-fold nested cross-validation was performed on the training set to search the optimistic parameters (*C*, from 10^−4^ ~ 10^3^), and the re-trained optimistic models were evaluated on the holdout testing set. The entire cross-validation process was repeated ten times. The all-relevant feature subsets from the repeated cross-validations were collected to determine the whole dataset's significantly essential predictors. We posed that the features were picked randomly as the null hypothesis. The ten iterations' expected frequency distributions were estimated by a binomial distribution B(p,N), where p was estimated as the mean fraction of selected features in the given modality's candidate features space, and *N* (=10) referred to the times of iterations. The significant ones were reported and discussed.

### 2.6. Performance Metrics

The main classification performance metrics, including AUC (area under the receiver operating characteristic curves), accuracy, sensitivity, specificity, and F1-score, are considered for evaluating the given diagnostic system. These metrics are originated from a confusion matrix. In our study's ambiance, condition positive samples are referred to subjects with ADHD, condition negative one's typical healthy subjects, prediction positive, and negative ones marked by the models. From the view of the models with a specific decision threshold, true-positive (TP) represents the count of subjects correctly labeled as ADHD, true-negative (TN) the count of ones correctly marked as TCs, and false-positive (FP) and false-negative (FN) the counts of misdiagnosis and missed diagnosis, respectively. Based on the definitions mentioned above, the metrics can be formulated as follows:


$$\begin{array}{l} accuracy=\frac{TP+TN}{TP+TN+FP+FN} \end{array}$$

$$\begin{array}{l} sensitivity=\frac{TP}{TP+FN} \end{array}$$

$$\begin{array}{l} specificity=\frac{TN}{TN+FP} \end{array}$$

$$\begin{array}{l} F1score=\frac{2\times TP}{2\times TP+FP+FN} \end{array}$$

Considering continuous decision thresholds from the strictest to the slackest, we can receive sequential pairs of sensitivity and specificity and plot the curve on the coordinate of 1 − *specificity* and *sensitivity*, the area under which is defined AUC. Accuracy, sensitivity, specificity, and F1-score are computed on the single probability cut-off = 0.5, while AUC, more comprehensive, evaluates the discriminative power under all thresholds. Therefore, we regarded AUC as the primary criterion of the models.

## 3. Results

This section will present the experimental results, including the selected features relevant to the ADHD disorder, the results of disorder diagnosis using the single modal and multimodal features and different fusion methods of multimodal features, and the effect of kernel weights for multiple kernel learning.

### 3.1. The Relevant Multimodal Features

**Figure 3:**
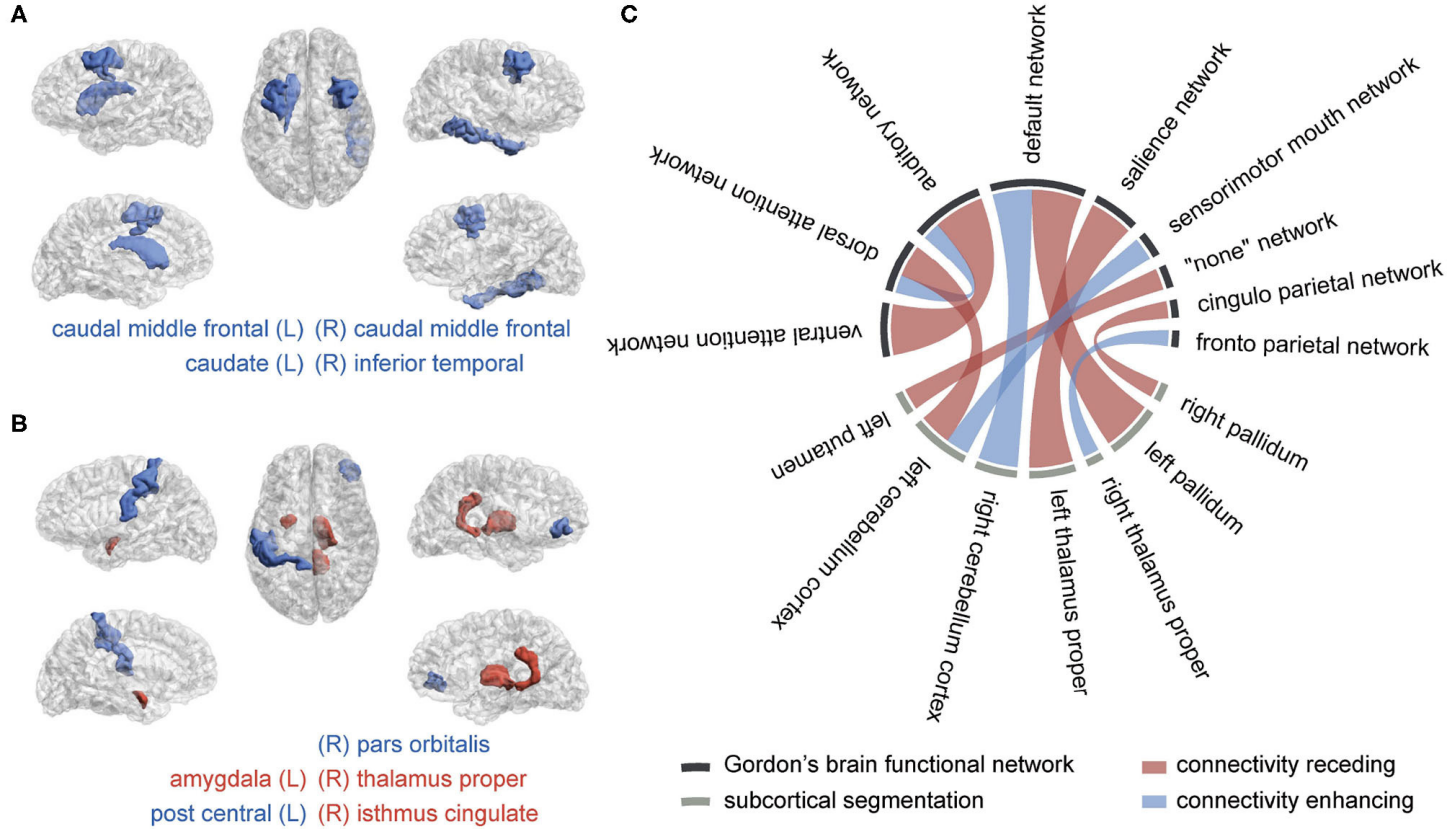
The brain regions and connectivity associated with attention-deficit/hyperactivity disorder (ADHD). **(A)** Macrostructural abnormal regions, **(B)** microstructural abnormal regions, and **(C)** abnormal functional connectivity The blue color represents a lessened measure in the ADHD group, while the red color represents a strengthened one. The more frequently they are picked in the selection procedure, the wider their bonds are.

The selected feature subset's average size was 4.7 (0.397% of all 1,184 features) for sMRI, 9.7 (0.821% of 1182) for DTI, and 12.8 (3.79% of 338) for rsfMRI shown as Figure 3A. With a *p*-value threshold of 0.05 (Holm–Bonferroni's multiple tests adjusted *p*-value, Holm, 1979), the features with significantly higher selection frequency were summarized, and we finally reported the intersection of significant features over ten independent experiments.

We obtained four significant features from sMRI, six from DTI, and ten from rsfMRI, listed in Table 2. Their distributions in two groups were plotted in Supplementary Figure 2, with the total selection frequencies and p-values between groups. The ROIs related to macrostructure and microstructure abnormalities were mapped to virtual brains from different perspectives, as shown in Figures 3A,B, respectively. The abnormal functional connectivity is shown in Figure 3C.

**Table 2:** Summarized predictors selected from multimodal MRI.

**Modality**	**Frequency (%)**	**ROI1**	**Type**	**Change**
*DTI*	81	Right pars orbitalis	FA	−
	81	Right isthmus cingulate	LD	+
	74	Left post central	FA	−
	69	Right isthmus cingulate	MD	+
	60	Left amygdala	MD	+
	58	Right thalamus proper	LD	+
*rsfMRI*	100	Ventral attention network and auditory network	Corr	+
	100	Left pallidum and default network	Corr	+
	91	Left thalamus proper and salience network	Corr	+
	86	Right cerebellum cortex and default network	Corr	−
	78	Left cerebellum cortex and dorsal attention network	Corr	+
	69	Left cerebellum cortex and sensorimotor mouth network	Corr	−
	67	Left putamen and “none” network	Corr	+
	64	Dorsal attention network and auditory network	Corr	−
	63	Right pallidum and cingulo parietal network	Corr	+
	62	Right thalamus proper and fronto parietal network	Corr	−
*sMRI*	66	Left caudal middle frontal	CA (mm^2^)	−
	62	Right caudal middle frontal	CV (mm^3^)	−
	62	Left caudate	T1w intensity	−
	52	Right inferior temporal	CV (mm^3^)	−

*Corr, average correlation between two ROIs; CA, cortical area; CV, cortical volume*.

### 3.2. Comparison of Unimodal and Multimodal Classification

**Figure 4:**
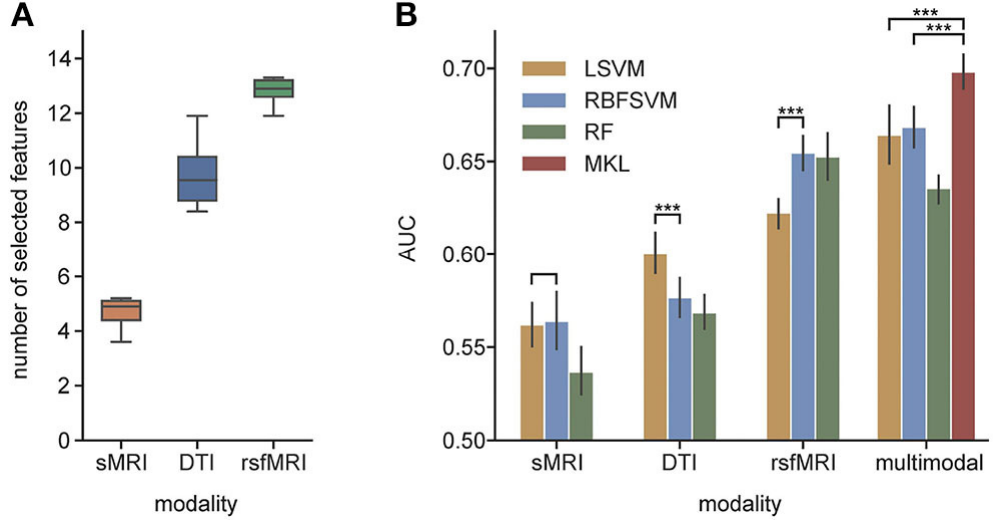
**(A)** The average number of selected features from each modality; **(B)** AUC of unimodal and multimodal classification. LSVM, linear kernel SVM; RBFSVM, radial basis function kernel SVM; RF, random forest; MKL, multiple kernel learning. ^***^Means that the *p*-value are less than 0.001.

In unimodal contexts, we built SVMs and RF to classify ADHD and TC. Resting-state MRI features achieved a significantly higher AUC (0.655) than the other two modalities. DTI features reached their own best AUC of 0.600, while sMRI features' best AUC was 0.564. We found the RBF kernel was suitable for FC, while the classification of DTI properties performed better on the linear kernel. Macrostructure features showed no significant preference between the linear kernel and RBF kernel. The RF classifiers did not perform better than any SVMs in unimodal contexts. We concatenated all selected predictors and fed them to the same SVMs and RF models as baseline fusion strategies, early fusion, of multimodal features. The AUC of SVM arises up to 0.668, but the RF classifier's performance dropped. The results are shown in Figure 4 and Table 3.

**Table 3:** Performances of unimodal and multimodal classification.

**Modality**	**Classifier**	**AUC**	**ACC**	**Sensitivity**	**Specificity**	**F1-score**
sMRI	LSVM	0.562	0.556	0.515	0.596	0.532
	RBFSVM	**0.564**	0.544	0.473	0.616	0.501
	RF	0.537	0.533	0.497	0.570	0.511
dMRI	LSVM	**0.600**	0.577	0.446	**0.710**	0.503
	RBFSVM	0.577	0.567	0.483	0.650	0.518
	RF	0.568	0.552	0.528	0.575	0.534
rsfMRI	LSVM	0.622	0.603	0.531	0.675	0.567
	RBFSVM	**0.655**	0.616	0.602	0.628	0.605
	RF	0.653	0.617	0.595	0.639	0.603
Multimodal	LSVM	0.664	0.629	0.565	0.696	0.598
	RBFSVM	0.668	0.611	0.599	0.623	0.602
	RF	0.636	0.596	0.590	0.602	0.590
	MKL	**0.698**	**0.643**	**0.609**	0.676	**0.626**

*The bold values are the best AUC in all unimodal settings and the globally best metrics*.

### 3.3. Comparison of Early Fusion and Kernel-Level Fusion

In the MKL framework, we fused the RBF kernel of sMRI features, a linear kernel of DTI features, and an RBF kernel of rsfMRI features. The AUC of MKL rose to 0.698, significantly higher than the early fusion strategies and any unimodal classifications. Besides the AUC, the accuracy and F1-score of MKL were 0.643 and 0.626, respectively, higher than any other context. The MKL's sensitivity and specificity were 0.609 and 0.676, which were better than most other classification settings but had limited improvement. Table 3 averaged the MKL frameworks' cross-validation performances of ten independent experiments. The statistically significant values were shown in Supplementary Table 4.

### 3.4. Effect of Kernel Weights for MKL-Based Multimodal Fusion

**Figure 5:**
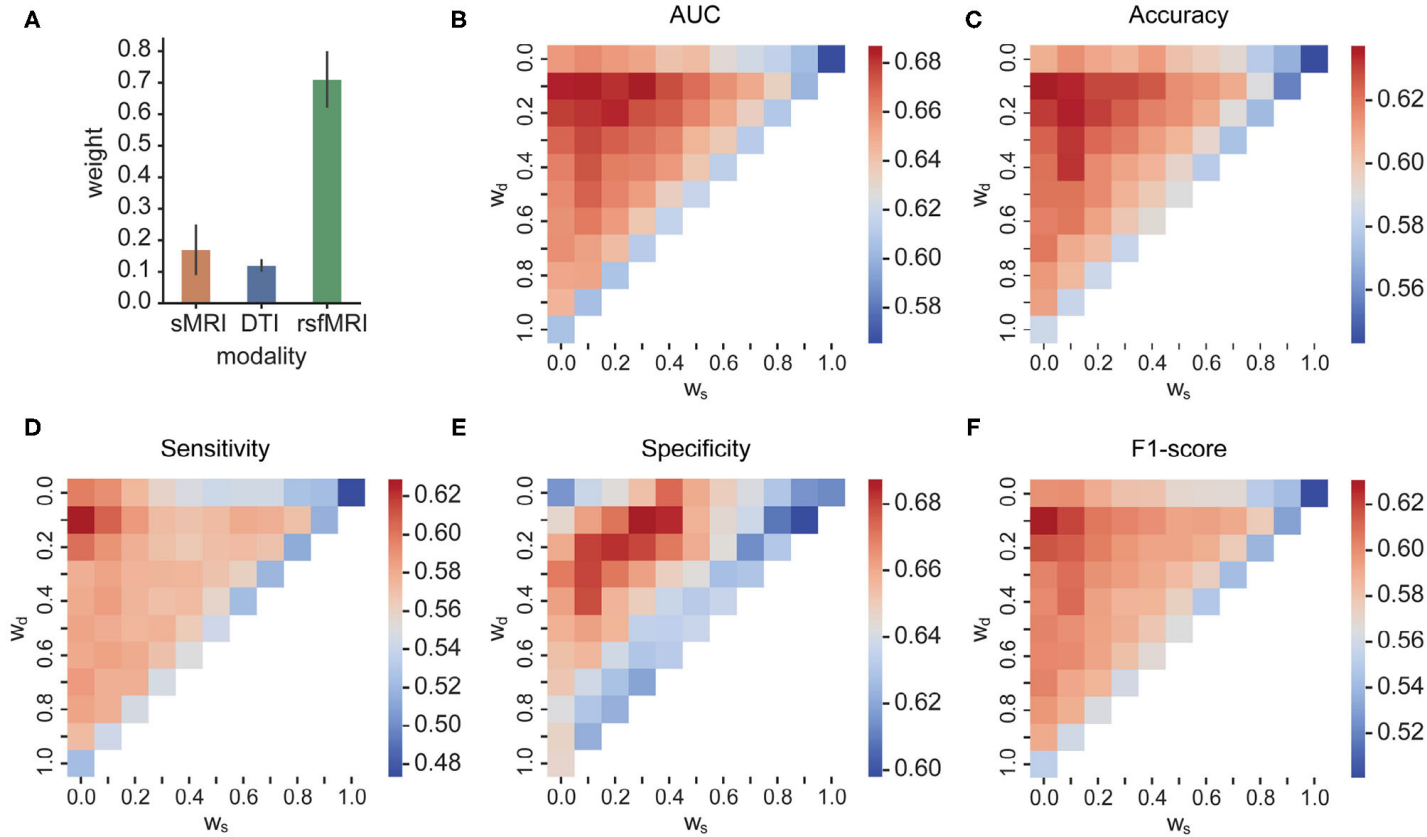
**(A)** The optimal weights of three modalities; **(B–F)** AUC, accuracy, sensitivity, specificity, and F1-score of different weight combinations in MKL, respectively. In each triangle, the vertices present the unimodality-based classification result, the top left rsfMRI, the top right sMRI, and the bottom left DTI. Similarly, the triangle edges between any two vertices display bimodal classifications with different weights.

The searched optimal weights assigned to the three modalities are plotted in Figure 5A. The predictors from rsfMRI have a significantly larger weight than sMRI and DTI predictors. The heatmaps in Figures 5B–F showed the main metric values of MKL concerning different weights combinations assigned to sMRI, DTI, and rsfMRI features. The shape of the heatmap is an upper triangle because of the constraint *w*_*s*_ + *w*_*rsf*_ + *w*_*d*_ = 1. The vertices present the unimodality-based classification result in each triangle, the top left rsfMRI, the top right sMRI, and the bottom left DTI. Similarly, the triangle edges between any two vertices display bimodal classifications with different weights. The results of bimodal and trimodal MKL classification are tabulated in Table 4. The trimodal setting achieved the best AUC, accuracy, and specificity, suggesting that every modality contributes to the classification indispensably. RsfMRI contributed the most from bimodal to trimodal classification. The fusion of sMRI + rsfMRI with DTI kernel significantly boosts the five metrics as well. However, the best F1-score and sensitivity reached their best on the bimodal fusion of rsfMRI and DTI, meaning that sMRI features have no extra contribution. The statistically significant values are shown in Supplementary Table 5.

**Table 4:** Performances of bimodal and trimodal multiple kernel learning (MKL) classification.

**Modality**	**AUC**	**ACC**	**Sensitivity**	**Specificity**	**F1-score**
sMRI + DTI	0.627	0.586	0.535	0.636	0.558
rsfMRI + sMRI	0.665	0.612	0.579	0.645	0.593
rsfMRI + DTI	0.690	0.639	0.630	0.648	0.632
rsfMRI + sMRI + DTI	0.698	0.643	0.609	0.676	0.626

## 4. Discussion

To the best of our knowledge, there is no other published study focusing on adolescent ADHD diagnosis based on the ABCD study. Our study proposed a kernel-level multimodal fusion and classification method for discriminating ADHD from the typical healthy controls. The kernel combination method assigned proper kernel functions and weights to the predictors from different modalities, including macrostructure properties, microstructure characters, and FC. The combined kernel can be naturally embedded into a typical SVM solver. The MKL shows better classification performance than unimodal or early fusion multimodal strategies on the ADHD image data from the ABCD study baseline year. Moreover, in the MKL framework, it is easy to quantify every modality's contributions, which are told by the optimal weights, propitious to explaining the result.

### 4.1. Support of Abnormality Findings

An RF-based all-relevant feature selection method, Boruta, was introduced to shrink the original feature space to discover all relevant brain image-based predictors for discriminating ADHD and TC. The predictors summarized in Figure 5 include four ROIs from macro-view, five ROIs from micro-view, and the connectivity related to nine cortical function networks and seven subcortical regions. The current literature can verify the anatomical regions and function networks. In our study, rsfMRI encodes the most information for distinguishing ADHD and TC. It achieves the best performance in unimodal testing and contributes to the most multimodal kernel for discriminating cases and controls. In the current literature, default mode network (DMN) (Cortese et al., 2012; Kucyi et al., 2015; Castellanos and Aoki, 2016), dorsal/ventral attention network (DAN and VAN) (Cortese et al., 2012; Kucyi et al., 2015; Castellanos and Aoki, 2016), sensorimotor network (SMN) (Cortese et al., 2012; Kucyi et al., 2015; Sörös et al., 2019), salience network (Sal) (Kucyi et al., 2015; Castellanos and Aoki, 2016), frontoparietal network (FP) (Cortese et al., 2012; Castellanos and Aoki, 2016), and auditory network (Aud) (Sörös et al., 2019) are the frequently reported abnormal FC in ADHD. On the other hand, the subcortical findings in connectivity, including pallidum (Castellanos and Aoki, 2016; Samea et al., 2019), thalamus (Bailey and Joyce, 2015), putamen (Cortese et al., 2012; Sörös et al., 2019), and cerebellum (Kucyi et al., 2015; Castellanos and Aoki, 2016), are in line with the existing literature, approved to play essential roles in the undergoing of ADHD. The DMN is selected most frequently in our study. As a network associated with task-irrelevant mental processes and mind wandering, DMN is usually suppressed when the subject is attending attention-needed external tasks. The abnormal hyperactivation of DMN will intrude on other task-related networks' functions, like attention and execution, and get the cognition distracted (Sonuga-Barke and Castellanos, 2007). Besides motor regulation, recent studies realized that the cerebellum intensively interacts in the activity, cognition, and emotion processes (Adamaszek et al., 2017; Sokolov et al., 2017; Schmahmann, 2019). We found the correlation between DMN and the cerebellum cortex gets decreased and diverging. Kucyi et al. has emphasized that the coupling between them, named CerDMN, plays an essential role in the cognition function mediated by cerebro-cerebellar interaction in ADHD (Kucyi et al., 2015). Moreover, the dysfunction within CerDMN can spread to other networks, including salience, dorsal attention, sensorimotor and frontoparietal networks (Kucyi et al., 2015). When it comes to the microstructural aspect, we found lower FA, which usually reflects axonal degeneration, in the right pars orbitalis parcellation. According to the prefrontal hypothesis of ADHD (Lin and Roth, 2017), the cerebral orbital region lesions associate with social disinhibition and impulse dyscontrol. We found the LD and MD increased in ADHD's right isthmus cingulate, a part of DMN, indicating the enhanced structural connectivity. The region associated with the left postcentral, located within SMN/Aud, has diminished FA value in ADHD. The MD was found to increase within ADHDs left amygdala, which was reported to receive inhibitory signals from the emotion control network (Dessel et al., 2020). As a transfer station connecting the cortex, basal ganglia, and cerebellum, the thalamus participates in modulating excitatory and inhibitory signals from both the ascending and descending pathways, which contributes to attention behaviors (Bailey and Joyce, 2015). We found the thalamus proper abnormality was related to both microstructure property (increased LD value) and FC with cortical networks (salience network and frontoparietal network). The area and volume decrease in the left caudal middle frontal of ADHD. This area was reported as the primary center of the frontal-striatum-thalamus circuit, responsible for cognitive and executive functioning (Audenaert et al., 2002) and associated with attention problems (Hoogman et al., 2019). The striatum, involving caudate, putamen, and pallidum, is regarded as a critical descending fiber bundle conducting the cerebral cortex and thalamus, maintaining muscular tension regulating fine motion. The recent study supported that striatum is involved in the hyperactivity pathogenesis of ADHD (Sörös et al., 2019).

It is interesting to notice the limited overlap of the ROIs summarized from the three modalities. For example, the basal ganglia regions, including pallidum, putamen, caudate, and thalamus, as well as the cerebellum, are frequently reported abnormal in ADHD in both macro-/micro-structure and function connection by existing literature (Greven et al., 2015; Castellanos and Aoki, 2016; Gehricke et al., 2017; Hoogman et al., 2017). However, we found that abnormal ROIs are more widespread in FC than structure. A study based on large-scale samples of ADHD reports that the cortical and subcortical structure abnormalities of ADHD are distinct across the lifespan (Hoogman et al., 2019). Given our study's relatively narrow age span, the misalignment might imply the asynchronous advancement of the macrostructural, microstructural, and brain functional abnormalities. Another possible reason is that FC is multi-linked by distinct nerve fiber tracts, which means there is no one-to-one relationship between FC and structural connectivity. The structural dysfunction of hub regions may lead to widespread functional connection anomalies. In our study, the coupling of FC predictors and structural, especially microstructural, predictors boost the ADHD diagnosis, suggesting the complementary advantage of multimodalities.

### 4.2. Effectiveness of MKL and Multimodal Features

The MKL framework achieves better performance than the single-kernel-based classifiers or RF classifiers that directly concatenate the features of all modalities. The kernel-level combination provides more flexibility to allocate distinct weights and kernel functions for individual modality. For example, string and categorical features cannot be concatenated with numeric features directly. Proper kernel functions can convert these heterogeneous features into comparable similarity scores (kernel matrix), which support weighted average. Like sMRI, dMRI, resting-state fMRI, task-based fMRI, CT, and electronic medical records, multiple data sources have become easily accessed these days in neuroscience. The kernel-based multimodal fusion method shows potentials in ADHD diagnosis.

In our experiment, rsfMRI drove the high performance, and the introduction of DTI brought significant classification improvement. However, the macrostructural features from sMRI did not show an impressive contribution, even though the kernels of sMRI and DTI shared comparable weights, which suggests FC coupled with microstructural property captures ADHD's profile in this age. The complicated relationship between functional and structural connectivity should be investigated further.

### 4.3. Comparison With Published Works

Up to now, most of the published works aiming at image-based ADHD diagnosis are based on ADHD-200. We chose several representative pieces of research for comparison. Their performances are listed in Table 5. We used additional features from DTI and outperformed the previous MKL method proposed by Dai et al. (Dai et al., 2012). However, our framework's performance only reached an above-average level in accuracy among the citations. These results could not illustrate that our MKL framework was inferior because we used a different dataset. The dataset of ADHD-200 has imbalanced demographic distribution between the cases and controls and the heterogeneity of ADHD diagnostic criteria and imaging sources (Milham et al., 2012). The winning team (Brown, 2012), from the University of Alberta, built the classifier on the phenotypic data of age, sex, handedness, and IQ and achieved the highest accuracy (62.5%) in this competition, even higher than image-based models or image-phenotype-mixed models, which has triggered discussion in the community about the usefulness of brain data in diagnosing a brain disorder (Brown, 2012; Arbabshirani et al., 2017). The brain's profile reflects the demographic divergence involving race, age, and gender. The underlying demographic/clinical differences between patients and health groups are believed to provide predictive power classification. However, the differences in demographic factors cannot be included in the diagnostic criteria. Meanwhile, the imbalance of the disease group's demographic distribution and control may cause false-positive findings that indicate the demography rather than mental diseases. The findings in these pieces of researches may originate from the imbalanced distribution of covariables between ADHD and its typical controls. Therefore, it is necessary to fix the covariables of demographic/clinical differences and look for more significant disease causes. In this study, we tried to cancel the covariable's intrinsic differences between groups to avoid false positives as much as possible. The batch effect of scanners was modeled and removed, which enabled the comparison among machines, and the main covariables were balanced between the groups, even though it may increase the classification difficulty.

**Table 5:** Attention-deficit/hyperactivity disorder (ADHD) classification results from published studies.

**Dataset**	**Modality**	**Classifier**	**AUC**	**ACC**	**Sensitivity**	**Specificity**	**F1-score**	**References**
ABCD	sMRI, rsfMRI, DTI	MKL	0.698	0.643	0.609	0.676	0.626	Our work
ADHD200	sMRI	Random Forest	–	0.754	–	–	–	(Wang et al., 2021)
ADHD200	sMRI, rsfMRI	CNN	–	0.692	–	–	–	(Zou et al., 2017)
ADHD200	sMRI, rsfMRI	linear kernel SVM	0.71	0.686	0.781	0.573	0.677	(Tan et al., 2017)
ADHD200	sMRI, rsfMRI	CNN+SVM	–	0.673	0.455	0.851	–	(Sen et al., 2018)
ADHD200	sMRI,	RBF kernel SVM	–	0.661	–	–	–	(Ghiassian et al., 2016)
ADHD200	rsfMRI	RBF kernel SVM	–	0.597	–	–	–	(Ghiassian et al., 2016)
ADHD200	personal character	linear kernel SVM	–	0.625	–	–	–	(Brown, 2012)
ADHD200	sMRI, rsfMRI	MKL	0.629	0.615	0.777	0.413	0.488	(Dai et al., 2012)
ADHD200	sMRI, rsfMRI	Kernel PCA	–	0.614	–	–	–	(Sidhu et al., 2012)
ADHD200	sMRI, rsfMRI	Random Forest	–	0.61	0.21	0.94	–	(Eloyan et al., 2012)
ADHD200	sMRI, rsfMRI, personal character	RBF kernel SVM	–	0.59	0.24	0.85	–	(Colby et al., 2012)
ADHD200	rsfMRI	Logistic regression	–	0.54	0.222	0.807	–	(Sato et al., 2012)

Recently, Owens et al. (2021) reported cross-validated elastic net regression to predict a continuous measure of ADHD symptomatology. Elastic net regression can simultaneously implement feature selection and regression by adding weighted L1/L2 regularized terms to the linear model's loss, limiting the scale of variable coefficients for avoiding overfitting. The models were built on morphometric profiles extracted from sMRI and brain region activation properties measured during three tasks of working memory, inhibitory control, and reward processing separately. The demographic, personal characteristic, and medical features were considered in the regression models as covariables. They observed a robust effect (*R*^2^ = 2%) of the working memory in predicting ADHD symptomatology and a dissipated impact (*R*^2^ = 1%) of the morphometric profiles when introducing covariables into the regression model. This group reported that they did not get robust models when utilizing the elastic net to predict the diagnosis from KSADS. It is worth noting that our study used more features than the published research and two modalities, DTI and resting-state fMRI, that they did not explore. We canceled the covariable effects by balancing the dataset and avoided overfitting by explicitly selecting all relevant features before prediction. The RF-based feature selection method shrinks the data dimension directly. It supports discovering the non-linear relationship between the features and target, but the linear regression model has no such capacity. Our SVM and MKL models achieved modest prediction performance of ADHD diagnosis, even though the weak discriminative power of sMRI features is partially consistent with this published work.

## 5. Limitation and Conclusion

Our study has several limitations that should be considered. We tried to diminish the influence of ADHD's comorbidities by simply removing the subjects suffering these, so the advantage of the large sample size cannot be fully taken. Our study has approved that the MKL method possesses the potentials to take full advantage of multimodal features, yet the moderate classification performance might be hard to apply in clinical diagnosis at the nonce.

These studies have approved that the features from distinct neuroimages modalities, including sMRI, DTI, and rsfMRI, encode complementary information for ADHD diagnosis, and kernel-level fusion could improve classification performance. The literature confirmed the identified multimodal predictors in the current study, and each modality provided specific importance in the MKL model, where the FC showed the most discriminative power.

## Data Availability Statement

Data used in the preparation of this article were obtained from the Adolescent Brain Cognitive Development (ABCD) Study (https://abcdstudy.org), held in the NIMH Data Archive (NDA). This is a multisite, longitudinal study designed to recruit more than 10,000 children age 9-10 and follow them over 10 years into early adulthood. The ABCD Study® is supported by the National Institutes of Health and additional federal partners under award numbers U01DA041048, U01DA050989, U01DA051016, U01DA041022, U01DA051018, U01DA051037, U01DA050987, U01DA041174, U01DA041106, U01DA041117, U01DA041028, U01DA041134, U01DA050988, U01DA051039, U01DA041156, U01DA041025, U01DA041120, U01DA051038, U01DA041148, U01DA041093, U01DA041089, U24DA041123, and U24DA041147. A full list of supporters is available at https://abcdstudy.org/federal-partners.html. A listing of participating sites and a complete listing of the study investigators can be found at https://abcdstudy.org/scientists/workgroups/. ABCD consortium investigators designed and implemented the study and/or provided data but did not necessarily participate in the analysis or writing of this report. This article reflects the views of the authors and may not reflect the opinions or views of the NIH or ABCD consortium investigators. The ABCD data repository grows and changes over time. The ABCD data used in this report came from version 2.0.1. DOIs can be found at https://nda.nih.gov/study.html?id=721.

## Author Contributions

XZ designed and implemented the multimodal ADHD classification pipeline, plotted the figures, and drafted this work. QL interpreted the found predictors related to ADHD. YG collected and interpreted the literature about ADHD. ZW undertook the statistical testing. HL and ML directed the experiment design and reviewed this article. All authors took part in the discussion of experiment design and edition of the article.

## Funding

This work was partly supported by the National Key R&D Program of China 2018YFC0910500, Neil Shen's SJTU Medical Research Fund, and Clinical Research Plan of SHDC (Grant NO. SHDC2020CR1047B).

## Conflict of Interest

The authors declare that the research was conducted in the absence of any commercial or financial relationships that could be construed as a potential conflict of interest.

## Publisher's Note

All claims expressed in this article are solely those of the authors and do not necessarily represent those of their affiliated organizations, or those of the publisher, the editors and the reviewers. Any product that may be evaluated in this article, or claim that may be made by its manufacturer, is not guaranteed or endorsed by the publisher.
