# Supplementary material for: Multimodal MR Images-Based Diagnosis of Early Adolescent Attention-Deficit/Hyperactivity Disorder Using Multiple Kernel Learning
Source: Front Neurosci. 2021 Sep 14;15:710133. doi: 10.3389/fnins.2021.710133 (PMC8477011; doi:10.3389/fnins.2021.710133)
Supplement: Supplementary file 2 [file Data_Sheet_1.PDF]

frontiers

Supplementary Material

1 IMAGE ACQUISITION AND PROCESSING

The standard scan session of ABCD Study includes sMRI series (T1w and T2w), dMRI series, rsfMRI series, and task-fMRI series for three tasks (MID, SST, and EN-back). Given the complexity of analysing task-based fMRI and explaining the result that stand beyond our capability, we considered sMRI, DTI (in dMRI series), and rsfMRI for our multimodal investigation. The T1w acquisition is a 3D T1w inversion prepared RF-spoiled gradient echo scan. On Siemens and GE scanners, the prospective motion correction was implemented, then the images were resampled to 1 mm isotropic. The T2w acquisition is a 3D T2w variable flip angle fast spin echo scan. The images were also corrected in prospective motion and resample to 1 mm isotropic. The dMRI acquisition (1.7mm isotropic) uses multiband EPI with slice acceleration factor 3 and includes 96 diffusion directions, seven $b = 0$ frames, and four b-values (6 directions with $b = 500s/mm^2$, 15 directions with $b = 1000s/mm^2$, 15 directions with $b = 2000s/mm^2$, and 60 directions with $b = 3000s/mm^2$). We only considered the diffusivity measures derived from the frames with b-values no more than $1000s/mm^2$ (referring to "inner shell fitting" in ABCD Study, release 2.0.1) to keep better correspond to those from traditional, single-b-value acquisition. The fMRI acquisitions ($2.4mm$ isotropic, $TR = 800ms$) also use multiband EPI with slice acceleration factor 6, and twenty minutes of resting state activity were collected. Each of the dMRI and fMRI acquisition blocks include fieldmap scans for B0 distortion correction. The imaging protocol was developed in collaboration with each scanner manufacturer using commercially available system upgrades, and where possible, product sequences. Imaging parameters were made as similar as possible across scanner manufacturers.

After image acquisition, the images of brain were aligned and parcellated with FreeSurfer (Fischl, 2012), version 5.3.0., using multiple atlases, which obtained corresponding numbers of regions of interest (ROIs). Multimodal measures (e.g., volume, intensity, mean beta weight, correlation between parcellation pairs) were estimated within these predefined ROIs. The ABCD team packaged the codes of processing pipeline and the complete execution environment to a Docker container, which can be downloaded from https://www.nitrc.org/projects/abcd_study.

All details of parameters and methods in image acquisition and processing were reported in previously published papers (Hagler et al., 2019; Casey et al., 2018).

2 REMOVING BATCH EFFECT VIA COMBAT

The Combat model (Johnson et al., 2007) was built to remove the batch effect from different scanners. It is a method widely applied in gene expression analysis, and recently, it has been proved to bear fruit in the context of radiomics, such as FA and MD extracted from DTI (Fortin et al., 2017), functional connectivity measurements extracted from fMRI (Yu et al., 2018), and cortical thickness extracted from T1w (Fortin et al., 2018). We defined the series number of every scanner as the batch factor, age as a numeric covariate, and sex/race as categorical covariates under our research condition. These factors are regarded to influence the actual values intrinsically. In order to estimate the batch effect as precisely as possible, we took all qualified subjects into the model. The batch effect is estimated in the linear model reformulated as Equation S1.

$$y_{ijf} = \alpha_f + \mathbf{x}_{ij}^T\beta_f + \gamma_{if} + \delta_i f\epsilon_{ijf} \tag{S1}$$

where y_{ijf} refers to the value of feature f measured in subject j from scanner i, and α_f is the average measure for feature f, $\mathbf{x}_ij$ the vector designed for covariates, and β_f the coefficients corresponding to x_ij. $\gamma_i f$ represents the additive effect related to scanner i, while $\delta_i f$ describes the multiplicative effect. The distribution of the error term ϵ_ijf was assumed following N(0,σ_v^2). The estimation of α_f, β_f, $\gamma_i f$ and $\delta_i f$ were symbolized as $\hat{\alpha}_f$, $\hat{\beta}_f$, $\hat{\gamma}_{if}$ and $\hat{\delta}_{if}$. Then, the modified measures y^*_{ijf} are given by Equation S2. We apply the python version of Combat matching the R version implemented in the sva package (Leek et al., 2012). Supplementary Figure S2 depicts the subjects t-SNE clusterings before and after removing the batch effect based on multimodal features.

$$y^*_{ijf} = \frac{y_{ijf} - \hat{\alpha}_f - \mathbf{x}_{ij}^T\hat{\beta}_f - \gamma_{if}}{\hat{\delta}_{if}} + \hat{\alpha}_f + \mathbf{x}_{ij}^T\hat{\beta}_f \tag{S2}$$

REFERENCES

Fischl B. Freesurfer. *NeuroImage* **62** (2012) 774–781. doi:10.1016/j.neuroimage.2012.01.021.

Hagler DJ, Hatton S, Cornejo MD, Makowski C, Fair DA, Dick AS, et al. Image processing and analysis methods for the adolescent brain cognitive development study. *NeuroImage* **202** (2019) 116091. doi:10.1016/j.neuroimage.2019.116091.

Casey B, Cannonier T, Conley MI, Cohen AO, Barch DM, Heitzeg MM, et al. The adolescent brain cognitive development (abcd) study: Imaging acquisition across 21 sites. *Developmental Cognitive Neuroscience* **32** (2018) 43–54. doi:10.1016/j.dcn.2018.03.001.

Johnson WE, Li C, Rabinovic A. Adjusting batch effects in microarray expression data using empirical bayes methods. *Biostatistics* **8** (2007) 118–127. doi:10.1093/biostatistics/kxj037.

Fortin JP, Parker D, Tunç B, Watanabe T, Elliott MA, Ruparel K, et al. Harmonization of multi-site diffusion tensor imaging data. *NeuroImage* **161** (2017) 149–170. doi:10.1016/j.neuroimage.2017.08.047.

Yu M, Linn KA, Cook PA, Phillips ML, McInnis M, Fava M, et al. Statistical harmonization corrects site effects in functional connectivity measurements from multi-site fmri data. *Human Brain Mapping* **39** (2018) 4213–4227. doi:10.1002/hbm.24241.

Fortin JP, Cullen N, Sheline YI, Taylor WD, Aselcioglu I, Cook PA, et al. Harmonization of cortical thickness measurements across scanners and sites. *NeuroImage* **167** (2018) 104–120. doi:10.1016/j.neuroimage.2017.11.024.

Leek JT, Johnson WE, Parker HS, Jaffe AE, Storey JD. The sva package for removing batch effects and other unwanted variation in high-throughput experiments. *Bioinformatics* **28** (2012) 882–883. doi:10.1093/bioinformatics/bts034.

3 SUPPLEMENTARY FIGURES

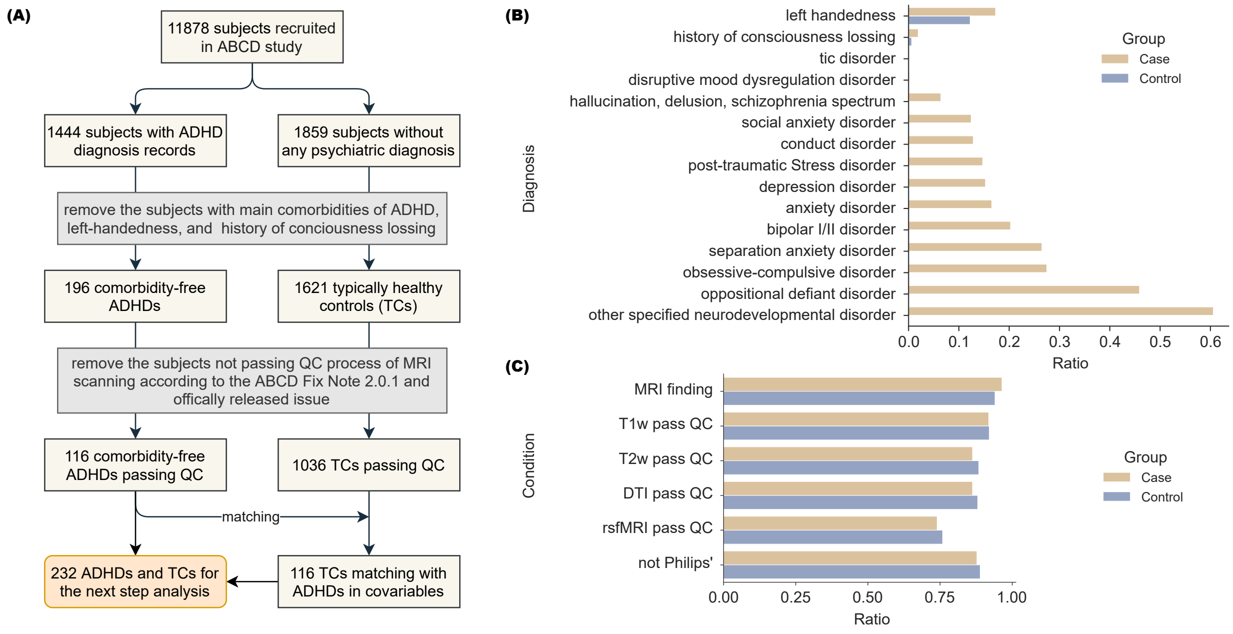

Figure S1. **(A)** Workflow of filtering samples based-on various exclusion criteria, showing the remained subjects after each major exclusion criteria; **(B)** comorbidity ratios in the both groups (to be excluded); **(C)** ratios of passing quality control process (to be included)

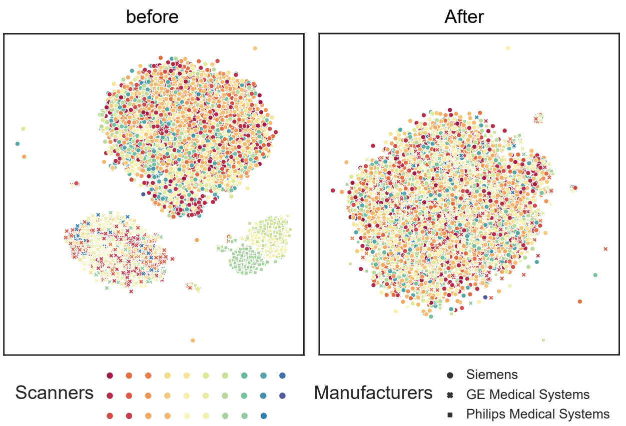

Figure S2. Subjects tSNE clustering before and after removing batch effect. The dots are colored by the corresponding scanners and shaped by the scanner's manufacturers. Before removing the batch effects, the aggregation indicates that every scanner has a unique systematic bias, and homogenization is in need. After the homogenization, the measures from all machines are corrected to the same scale and become comparable.

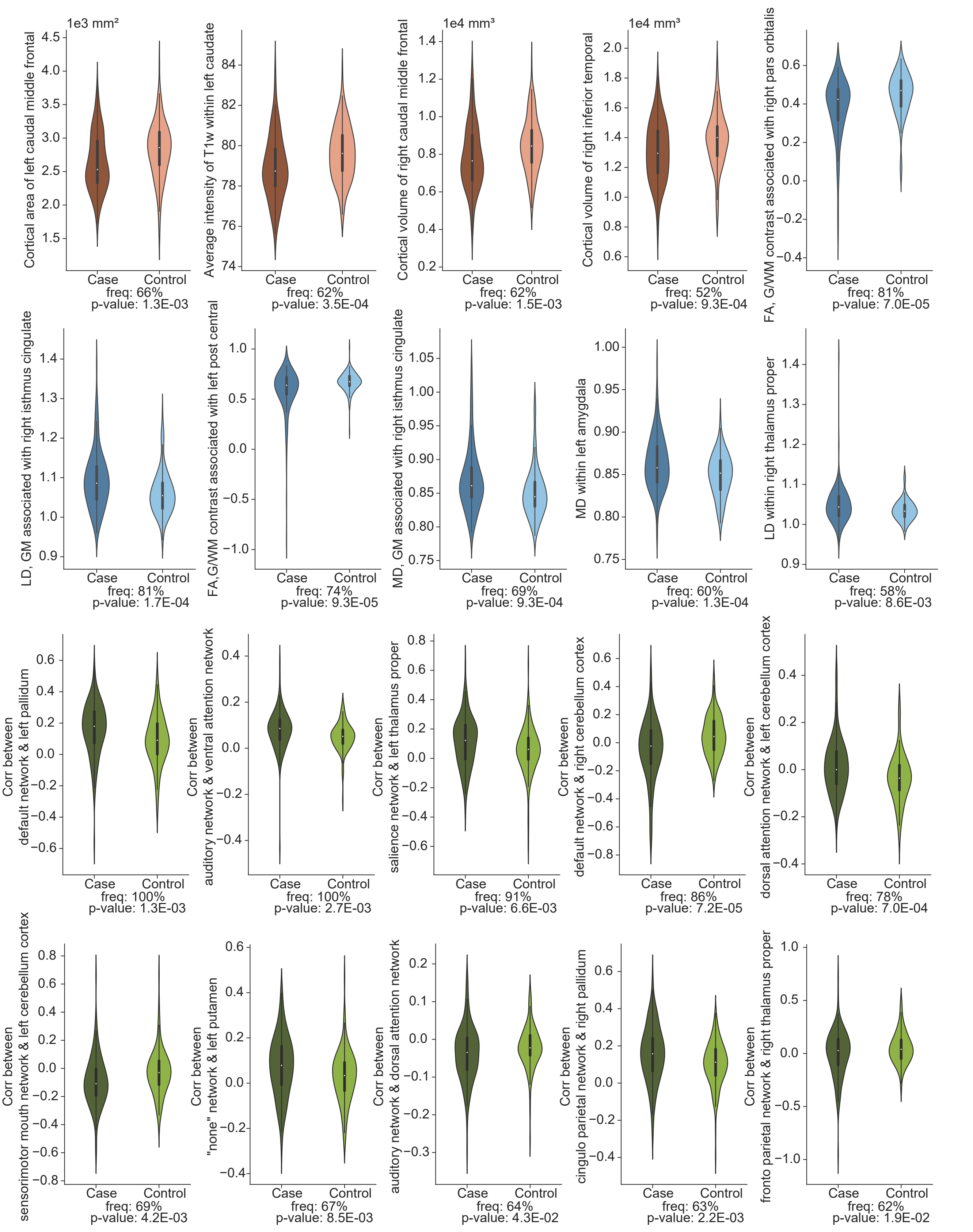

Figure S3. The distribution difference of summarized predictors of macrostructure, microstructure, and functional connectivity. The hue of orange represents macrostructural properties, blue microstructural properties, green functional connectivity.
